# Charcot-Leyden crystals in a prostatic adenocarcinoma

**DOI:** 10.1186/1746-1596-1-26

**Published:** 2006-09-07

**Authors:** Özgür Aydın

**Affiliations:** 1 Alanya Hospital, Department of Pathology, Başkent University, Alanya, Antalya, Turkey

## Abstract

A transrectal needle biopsy from a 63-year-old man was decided because of a high prostatic spesific antigen in the blood, and a hard right lobe in rectal examination. 10 examples were taken from each lobe. In 1 of 4 of the examples from the left lobe, which contained a small focus of adenocarcinoma, numerous eosinophils were observed to surround the carcinomatous focus and attack the carcinoma cells. Uniquely, at the same focus Charcot-Leyden crystals could be seen in the intraluminal space and stromal area. A carcinoma oriented eosinophil accumulation, and Charcot-Leyden crystals in prostate was not described before.

A transrectal needle biopsy was indicated due to a high level of prostatic spesific antigen in the blood, and a hard right lobe in rectal examination of a 63-year-old man. 10 examples were taken from each lobe of the prostate. In microscopic examination, all examples from the right lobe contained an adenocarcinoma covering 25 to 50% of the biopsies. Only 4 of the examples from the left lobe contained the tumor, which were all small focuses, composed of 8–10 glands. It was one of the biopsies from the left lobe with a small group of neoplastic glands that attracted our attention. Initially, it was the intraluminal bright, eosinophilic crystalloids as a striking low-power appearance. In daily practice, finding crystalloids in a transrectal needle biopsy with a prostatic adenocarcinoma should not be so astonishing. On the contrary, demonstration of intraluminal crystalloids is common in such cases, and even perceived to be an aid in the diagnosis of prostatic adenocarcinoma [1]. The neoplastic group was also incorporated with a dense infiltration of eosinophils. A carefull re-evaluation of other biopsies confirmed that the feature was peculiar to the aforementioned focus. The reaction, which seemed to be almost totally composed of eosinophils, was clearly restricted to the carcinomatous glandular group. Eosinophils were not only surrounding the focus but also looked like 'attacking' the carcinoma cells. Intraepithelial eosinophils forming occasional small groups could easily be found. The interglandular stroma was rich in free granules, an absolute evidence of eosinophil activation. The co-existance of the eosinophils and the crystalloids certainly pointed to a relationship between two. Actually, the crystalloids were extremely similar to the Charcot-Leyden crystals, that we were familiar with in many allergic situations. It was not unlikely, as these crystals were considered to be an other morphologic hallmark of eosinophil activation. When searched, the crystals could also be found in the stroma.

Charcot-Leyden crystals are known to be composed of a single protein, released by the activated eosinophil, called lysolecithin acylhydrolase, which has lysophospholipase activity. Lysolecithin acylhydrolase is one of several eosinophil proteins with cytotoxic properties involved in the eosinophil's antiparasitic, antineoplastic, and immune functions [2]. In this case, co-existing eosinophil accumulation, degranulation and extraluminal presentation gave us confidence to call the bodies as Charcot-Leyden crystals. Morphological and histochemical searches indicate that the intraluminal contents (all crystalloids, corpora amylacea, and others) in benign and malignant glands form a continuous spectrum and are largely composed of material derived from the components of lining cells [3]. Naturally, they are observed as glandular intraluminal contents.

The homogenous nature of the infiltration suggested that such an infiltration might be induced by a tumor-derived eosinophil chemotactic factor. An explanation for this could be an extra-step mutation that caused a release of the stimulating factor that enhanced eosinophil chemotaxis.

The point is; this isolated focus, which did not show any histomorphologic difference from the other neoplastic tissue, was subject to an "selective" attemp of destruction by a spesific subgroup of the cellulary host defence. This feature might be inspiring in developing approaches towards enhanced management strategies for cancer treatment [4].

**Figure 1:**
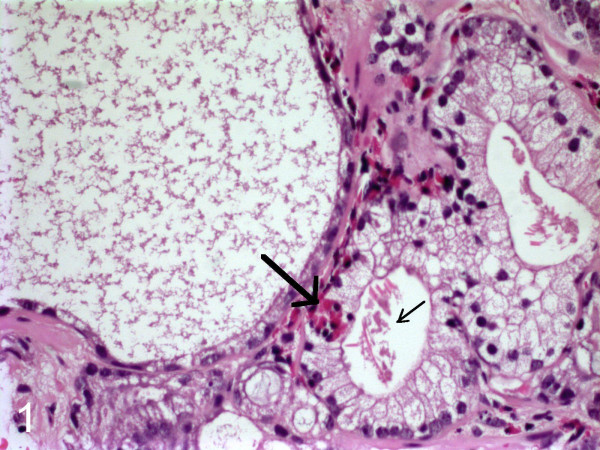
The peritumor fibrous stroma contained degranulated eosinophils. An intraepithelial group of eosinophils is seen (thick arrow). Charcot-Leyden crystals were seen intraluminally (thin arrow).

**Figure 2:**
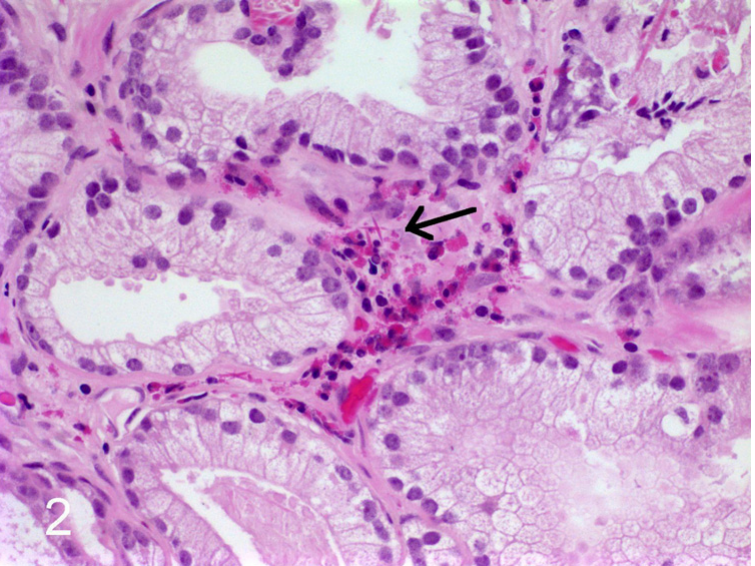
The crystals (arrow) could also be found in the stroma.
